# Testing the additional predictive value of high-dimensional molecular data

**DOI:** 10.1186/1471-2105-11-78

**Published:** 2010-02-08

**Authors:** Anne-Laure Boulesteix, Torsten Hothorn

**Affiliations:** 1 Department of Medical Informatics, Biometry and Epidemiology, University of Munich, Marchioninistr, 15, D-81377 Munich, Germany; 2 Department of Statistics, University of Munich, Ludwigstr 33, D-80539 Munich, Germany

## Abstract

**Background:**

While high-dimensional molecular data such as microarray gene expression data have been used for disease outcome prediction or diagnosis purposes for about ten years in biomedical research, the question of the additional predictive value of such data given that classical predictors are already available has long been under-considered in the bioinformatics literature.

**Results:**

We suggest an intuitive permutation-based testing procedure for assessing the additional predictive value of high-dimensional molecular data. Our method combines two well-known statistical tools: logistic regression and boosting regression. We give clear advice for the choice of the only method parameter (the number of boosting iterations). In simulations, our novel approach is found to have very good power in different settings, e.g. few strong predictors or many weak predictors. For illustrative purpose, it is applied to the two publicly available cancer data sets.

**Conclusions:**

Our simple and computationally efficient approach can be used to globally assess the additional predictive power of a large number of candidate predictors given that a few clinical covariates or a known prognostic index are already available. It is implemented in the R package "globalboosttest" which is publicly available from R-forge and will be sent to the CRAN as soon as possible.

## Background

While high-dimensional molecular data such as microarray gene expression data have been used for disease outcome prediction or diagnosis purposes for about ten years [1] in biomedical research, the question of the additional predictive value of such data given that classical predictors are already available has long been under-considered in the bioinformatics literature.

This issue can be summarized as follows. For a given prediction problem (for example tumor subtype diagnosis or long-term outcome prediction), we consider two types of predictors. On the one hand, conventional clinical covariates such as, e.g. age, sex, disease duration or tumor stage are available as potential predictors. They have often been extensively investigated and validated in previous studies. On the other hand, we have molecular predictors which are generally much more difficult to measure and collect than conventional clinical predictors, and not yet well-established. In the context of translational biomedical research, investigators are interested in the additional predictive value of such predictors over classical clinical covariates.

A particular challenge from the statistical point of view is that these molecular predictors are often high-dimensional, which potentially leads to overfitting problems and overoptimistic conclusions on their additional predictive power [2,3]. The question whether high-dimensional molecular data like microarray gene expression have additional predictive power compared to clinical variables can thus not be answered using standard statistical tools such as logistic regression (for class prediction) or the proportional hazard model (for survival analysis). Hence, there is a demand for alternative approaches.

The formulation "additional predictive value compared to classical clinical predictors" is ambiguous because it actually encompasses two distinct scenarii. In the first scenario, the prediction model based on clinical covariates is given (for instance from a previous publication) and can be directly applied to the considered data set. Such models are usually denoted as "risk score" or "index" in the medical literature and often use a very small number of predictors, such that they are widely applicable in further studies. However, clinicians often want to develop their own clinical score using their own data (second scenario) because it is expected to yield higher accuracy for their particular patient collective, or because they want to predict a different outcome or use different predictors. These two scenarii are different from the statistical point of view: in the first scenario the prediction rule based on clinical covariates is fixed, while it has to be constructed from the data in the second scenario.

In this article, we present a method for testing the additional predictive value of high-dimensional data that fulfills the following prerequisites:

• **Prerequisite 1**: The additional predictive value is assessed within a hypothesis testing framework where the null hypothesis corresponds to "no additional predictive value".

• **Prerequisite 2**: The focus is on the *additional *predictive value, i.e. the model selection procedure for the high-dimensional data takes the clinical covariates into account.

• **Prerequisite 3**: The method can address the two scenarii described above (fixed risk score or clinical prediction model estimated from the data).

Note that our aim is *not *to construct a combined prediction rule based on clinical and high-dimensional data: the focus is on the testing aspect.

In the last few years, a couple of methods fulfilling one of these three prerequisites have been proposed to handle this problem. In the context of class prediction, the pre-validation procedure proposed by Efron and Tibshirani [4,5] consists of constructing a prediction rule based on the high-dimensional molecular data only within a cross-validation framework. The cross-validated predicted probabilities are then considered as a new pseudo-predictor. The question of the additional predictive value is answered by classical hypothesis testing within a logistic regression model involving both the clinical covariates and the cross-validated predicted probabilities. However, this approach may yield a substantial bias because, roughly speaking, the cross-validated probabilities are not independent from each other. This bias is quantitatively assessed in the subsequent publication [5]. The authors suggest a (computationally intensive) permutation-based testing scheme to circumvent this problem. Another pitfall of the pre-validation procedure is that the cross-validated probabilities are constructed without taking the clinical covariates into account. Hence, pre-validation does not fulfill prerequisite 2. For example, if the high-dimensional molecular predictors are highly correlated with the clinical predictors, so will be the cross-validated predicted probabilities. Constructing the cross-validated predicted probabilities in such a way that they are complementary to rather than redundant with the clinical covariates potentially yields different results [6]. On one hand, pre-validation as originally suggested [4] may overestimate the additional predictive value because the predictive value of clinical covariates is "shared" by the clinical covariates themselves and the cross-validated predicted probabilities in the logistic regression model, due to correlation. On the other hand, it may be underestimated because subtle contributions of the high-dimensional molecular data to the prediction problem are likely to be overcome by more obvious contributions- which are redundant with the contributions of the clinical covariates.

Another important method for assessing high-dimensional predictors while adjusting for clinical covariates is Goeman's global test [7]. In the generalized linear model framework, it is assumed that the regression coefficients of the molecular variables are sampled from some common distribution with expectation zero and variance *τ*^2^. The null-hypothesis that all regression coefficients are zero can then be reformulated as *τ*^2 ^= 0. In their second paper on this subject, the same authors suggest a variant of this test that adjusts for additional (e.g. clinical) covariates in the context of survival analysis [8]. This adjustment methodology can also be applied to the case of class prediction and is implemented in the function globaltest from the Bioconductor package **globaltest **[9] through the adjust option. In the present paper, we address this question using a completely different methodology based on permutation testing and boosting regression. Other authors address the issue of the additional predictive value in the context of prediction and derive combined prediction rules using both clinical predictors and high-dimensional molecular data. A method proposed recently embeds the pre-validation procedure described above into PLS dimension reduction and then uses both clinical covariates and pre-validated PLS components as predictors in a random forest [10]. This method has the same inconvenience as the original pre-validation approach, in the sense that the PLS components are built without taking the clinical covariates into account. They may thus be redundant with clinical predictors and do not focus particularly on the residual variability, as outlined above for the original pre-validation procedure. Hence, this method does not fulfill prerequisite 2. This pitfall is shared by many recent machine learning approaches for constructing combined classifiers using both clinical and high-dimensional molecular data [11,12].

In contrast, the CoxBoost approach [6] for survival analysis with mandatory covariates takes clinical covariates into account while selecting the model for the high-dimensional predictors. Clinical covariates are forced into the model through a customized penalty matrix. The authors suggest to set this penalty matrix to a diagonal matrix with entries 1 and 0 for "penalization" and "no penalization", respectively. This approach has the major advantages that it can i) take into account the clinical covariates while updating the coefficients of the molecular variables, ii) easily handle the *n *≪ *p*, and iii) yield a sparse molecular signature without additional preliminary variable selection procedure. The CoxBoost approach is presented as a survival prediction method. However, a similar procedure can be used in the context of class prediction [13]. This approach fulfills prerequisite 2 but not prerequisite 1 since its aim is to provide a combined prediction model rather than a testing procedure.

Motivated by the strong advantages of the CoxBoost approach, we suggest an alternative simple two-stage approach which also uses a boosting algorithm, but in a different scheme which is more appropriate for the testing purposes considered here. Our approach combines a standard generalized linear model for modeling the clinical covariates (step 1) with a boosting algorithm for modeling the additional predictive value of high-dimensional molecular data (step 2). The differences between our approach and the CoxBoost approach [6] are as follows. In contrast to the CoxBoost method, we first fit a classical generalized linear model to the clinical covariates (first step) and then focus on the molecular variables (second step) without changing the coefficients fitted in the first step. This makes our procedure potentially easier to interpret, since most clinicians are familiar with standard logistic regression or Cox regression which are used in the first step but might be confused by the iterative update of the coefficients. Moreover, by fixing the coefficients of the clinical covariates in the first step, we set the focus on additional predictive value more clearly than if these coefficients are allowed to change depending on the effect of the molecular variables. Moreover, we follow the well-established boosting algorithm described in [14] in which the update *g*^[*m*] ^(see 'Methods' Section for an explanation of the notation) is multiplied by a small shrinkage factor *ν*. Instead, CoxBoost does not multiply by *ν *but penalizes the update through a penalty matrix in the loss function. Like the CoxBoost approach, our method fulfills prerequisite 2. To address prerequisite 1, we suggest a simple permutation-based testing procedure. The resulting novel approach thus fulfills the two first prerequisites. Moreover, we suggest a variant for addressing the application of a risk score fitted previously using other data (prerequisite 3).

In the next section, we briefly review the methods involved in the first step (logistic regression) and second step (boosting with componentwise linear least squares), and we describe the combined two-step procedure as well as the permutation test.

## Methods

In the following, we consider a random vector of clinical covariates (*Z*_1_,..., *Z*_*q*_)^⊤ ^with *n *independent realizations ***z***_*i *_= (*z*_*i*1_,..., *z*_*iq*_)^⊤^, for *i *= 1,..., *n*. Similarly, the random vector of molecular covariates is denoted as (*X*_1_,..., *X*_*p*_)^⊤ ^(with *p *>*n*) with *n *realizations ***x***_*i *_= (*x*_*i*1_,..., *x*_*ip*_)^⊤^, for *i *= 1,..., *n*. The response variable is denoted as *Y *and coded as *Y *∈ {-1, 1}, with realizations *y*_1_,..., *y*_*n*_.

### Logistic regression

Logistic regression is the standard statistical tool for constructing linear class prediction rules and assessing the significance of each predictor. It is implemented in all statistical software tools, for instance in R within the generic function glm. The logistic regression model is given as(1)

where *Y *is the binary response variable of interest and *Z*_1_,..., *Z*_*q *_denote the *q *predictors. In the two-stage approach suggested in this article, *Z*_1_,..., *Z*_*q *_correspond to the clinical predictors. The maximum-likelihood estimates  of the model coefficients *β*_0_,..., *β*_*q *_can be obtained via iterative algorithms such as the Newton-Raphson procedure. For each new observation ***z***_new _= (*z*_new,1_,..., *z*_new, *q*_)^⊤^, one obtains the so-called *linear predictor *as(2)

from which the predicted probability  is derived as . In our two-stage approach, the estimated logistic regression coefficients  of the clinical covariates which are fitted in the first step are passed to the second step that uses the corresponding linear predictor as an offset.

### Boosting with componentwise linear least squares

#### General algorithm

In this section, we give a short general overview of boosting as reviewed by Bühlmann and Hothorn [14], and explain which variant of boosting we use in the second step of our two-stage procedure. The considered predictors are the molecular covariates *X*_1_,..., *X*_*p*_. The AdaBoost algorithm was originally developed by Freund and Schapire as a machine learning tool, see [15] for an early reference. Friedman, Hastie and Tibshirani [16] then developed a more general statistical framework which yields a direct interpretation of boosting as a method for function estimation. The goal is to estimate a real-valued function(3)

where *ρ*(·) is a loss function which will be discussed in this section. Friedman, Hastie and Tibshirani [16] formulate boosting as a functional gradient descent algorithm for estimating *f*(·) as sketched below [14].

1. Initialize (·) with an offset value, for instance (·) = 0 or . Set *m *= 0.

2. Increase *m *by 1. Compute the negative gradient *ρ*(*Y, f*) and evaluate it at (***x***_*i*_), for each observation *i *= 1,..., *n*:(4)

3. Fit the *u*_1_,..., *u*_*n *_to ***x***_1_,...,***x***_*n *_using a so-called base procedure (which will be discussed later in this section):(5)

4. Update , where 0 <*ν *≤ 1 is a step-length factor (see below), that is, proceed along an estimate of the negative gradient vector.

5. Iterate steps 2 to 4 until *m *= *m*_stop _for some stopping iteration *m*_stop_.

Note that the offset term is simply the best constant model (without taking the covariates into account) and, therefore, the algorithm starts at the center of the unconditional distribution of the response for fitting the conditional distributions.

#### The boosting version used in the present study

In the context of binary class prediction (i.e. when *Y *is binary), it is usual to use the so-called log-likelihood loss function(6)

in step 2 [14]. In the present study, we stick to this standard choice which yields nice properties. For instance, it can be shown that the population minimizer of this loss function has the intuitive form .

In order to fit a model which is linear in the molecular variables, componentwise linear least squares regression is applied as an efficient base procedure in step 3. This base procedure is defined as(7)

where  simply denotes the least square estimate of the coefficient *β*_*j *_in the univariate regression model including *X*_*j *_as single predictor(8)

and j* corresponds to the predictor yielding the best prediction in this univariate regression model:(9)

Meanwhile, componentwise linear least squares can be considered as one of the standard base procedures for boosting. We choose it as a base procedure for the second step of our two-stage analysis scheme. A major advantage of componentwise linear least squares as a base procedure in the context of our two-stage approach is that the final estimated function (·) can be seen as a linear combination of the molecular predictors *X*_1_,..., *X*_*p *_of the same form as the linear combination of the clinical covariates *Z*_1_,..., *Z*_*q *_output by the first step. Hence, it is easy to combine both steps of the analysis, as explained in the Section 'Combining logistic regression (step 1) and boosting (step 2)'.

### Combining logistic regression (step 1) and boosting (step 2)

In this section, we show how logistic regression and boosting as described in the two above sections can be combined into a two-step procedure. We first present the procedure for the case when the model with clinical covariates has to be estimated from the data and then address the other scenario (application of a fixed risk score known from a previous study).

#### Step 1

1.1 Fit a logistic regression model as outlined in the Section 'Logistic regression' to the clinical covariates *Z*_1_,..., *Z*_*q*_, yielding estimates  for the logistic regression coefficients.

1.2 Compute the linear predictor  for *i *= 1,..., *n*.

#### Step 2: Boosting regression

This step involves one method parameter, the number of boosting iterations *m*_stop_, which is discussed in the Section 'The choice of *m*_stop_'.

2.1 Define the offset function (·) as  and run the boosting algorithm given in the Section 'Boosting with componentwise linear least squares' using the log-likelihood loss function *ρ*_log-lik _and componentwise linear least squares as a base procedure with *m*_stop _boosting iterations, as implemented in the R package **mboost **[17,18]. Derive the estimates  for the intercept and the regression coefficients of the variables *X*_1_,..., *X*_*p*_. Note that, in practice, many of these coefficients are zero.

2.2 Compute the resulting linear predictor as(10)

2.3 Compute the predicted probabilities from the linear predictor as  and derive the average negative binomial log-likelihood as(11)

A small negative binomial log-likelihood indicates good model fit. Note that we could have used another goodness criterion in place of the negative binomial log-likelihood. However, the binomial log-likelihood is especially appropriate, since it is the criterion optimized by the boosting procedure. To assess the additional predictive value of the molecular data, we suggest to compare ℓ to the negative binomial log-likelihood obtained from permuted data, as outlined in the Section 'Permutation-based testing procedure'.

In the situation where a risk score is already available (e.g. from a previous publication), step 1 can be skipped. The linear predictor  is obtained through logit transformation of the risk score and used as an offset in boosting regression in place of the estimated linear predictor . Our method can thus accommodate situations where the clinical risk score is not based on a linear predictor in the context of logistic regression (for instance a risk score corresponding to a classiffication tree).

Alternatively, our method can also be used to globally assess the molecular variables independently of any clinical covariates. This would be done by ignoring the first step (logistic regression) of our method and simply setting the offset to the value of the intercept.

### Permutation-based testing procedure

We consider the null-hypothesis that the variables *X*_1_,..., *X*_*p *_have no additional predictive power given the clinical covariates. The considered model is given as(13)

and the null-hypothesis is formally stated as(14)

We suggest to test this null-hypothesis using a permutation procedure by permuting *X*_1_,..., *X*_*p *_only. More precisely, we replace ***x***_1_,...,***x***_*n *_by ***x***_*σ*(1),...,_***x***_*σ*(*n*)_, where *σ *is a random permutation of (1,..., *n*), while the clinical covariates ***z***_*i *_are not permuted. The two-step procedure is applied and the negative binomial log-likelihood ℓ is computed again for this permuted data set. The whole procedure is repeated a large number of times *B*, yielding the negative binomial log-likelihoods ℓ_1_,...,ℓ_*B*_. The permutation *p*-value is then obtained as(15)

where **1 **denotes the indicator function.

In the case of a fixed risk score as discussed at the end of the Section 'Combining logistic regression (step 1) and boosting (step 2)', the underlying model is slightly different and can be formulated as(16)

where *R*(.) denote the fixed risk score function based on the clinical covariates *Z*_1_,..., *Z*_*q*_. Note that, if *R*(.) is simply a linear function of *Z*_1_,..., *Z*_*q*_, this version of the test will rather lead to rejection of the null-hypothesis than the first version with coefficients *β*_1_,..., *β*_*q *_estimated from the data.

### The choice of *m*_stop_

When boosting is used for building a prediction model, the choice of the number of boosting iterations is crucial. A too large *m*_stop _would yield an overcomplex model overfitting the training data, while a too small *m*_stop _would yield a too sparse model that do not fully exploit the available predicting information. In practice, the number of boosting iterations can be selected using an AIC-like criterion or by minimization of the out-of-sample negative binomial likelihood within a bootstrap procedure [14]. In contrast to what happens in the context of prediction, the results of our approach for the assessment of additional predictive value are not strongly affected by the number of boosting iterations. For large values of *m*_stop_, the obtained regression model overfits the training data set, but the differences between permuted and non-permuted data, on which the test is based, do not seem to strongly depend on the number of boosting steps.

**Figure 1:**
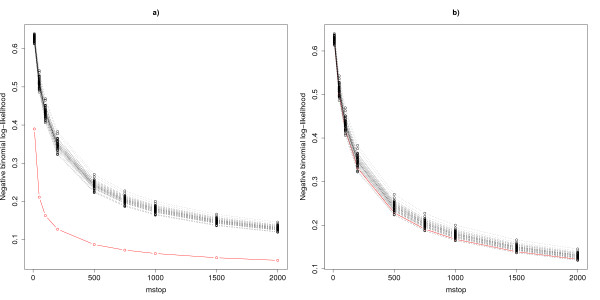
**Choice of *m*_stop_**. Negative log-likelihood for the original data (red) and the permuted data (black) against the number of iterations *m*_stop_. (a) *μ*_*X *_= 5, p* = 1. (b) *μ*_*X *_= 0.2, p* = 200.

To illustrate this, we follow the simulation scheme described in the 'Results' section and consider two extreme cases: a) one strongly informative molecular variable (*μ*_*X *_= 5, p* = 1) and b) 200 very weakly informative molecular variables (*μ*_*X *_= 0.2, p* = 200), all the other molecular variables and clinical covariates being irrelevant for the prediction problem. The second setting can be considered as an extreme case, since there are often less than 200 informative variables in practice, and relevant between-group shifts are often larger than *μ*_*X *_= 0.2. In these settings, we compute the negative binomial log-likelihood ℓ as well as its permuted versions ℓ_1_,...,ℓ_*B *_for a grid of *m*_stop _values ranging from 10 to 2000. The resulting curves are displayed in Figure 1. Similar curves are obtained for different values of the simulation parameters. To sum up, the curve of the original data set (with informative *X *variables) decreases with increasing *m*_stop _more rapidly than the curves of the permuted data sets until a certain value of *m*_stop_. After this value, all curves are approximately parallel. Hence, further increasing *m*_stop _would not change the test result much. This is because, roughly speaking, the newly added components do not improve the model anymore - even with the original non-permuted variables.

As an objective criterion, we suggest to choose the *m*_stop _value based on the AIC procedure described by Bühlmann and Hothorn [14]. The only remaining parameter is then the maximal number of boosting iterations . Except from the computational expense, there is no inconvenience to choose a very large value, for example  = 1000.

### Computational cost

The computation time grows linearly with the number of boosting regressions, i.e. the number of permutations. For usual data sets such as those considered in this paper, boosting regression runs in less than one second with a standard PC (Intel(R) Core(TM)2 CPU T7200 2.00 GHz). Note that the permutation-based procedure can be parallelized very easily, since the permutations are independent of each other.

## Results

### Simulation design

In all settings, the number *n *of observations is set to *n *= 100, the number *p *of molecular predictors to *p *= 1000 and the number *q *of clinical predictors to *q *= 5. The binary variable *Y *is drawn from a Bernoulli distribution with probability of success 0.5. The p* relevant molecular variables follow the conditional distribution *X*_*j*_|(*Y *= 1) ~ (*μ*_*X*_, 1) and *X*_*j*_|(*Y *= -1) ~ (0, 1), for *j *= 1,..., p*. The other molecular variables *X*_p*+1_,..., *X*_*p *_simply follow a standard normal distribution. Similarly, the clinical covariates are drawn from the conditional normal distribution *Z*_*j*_|(*Y *= 1) ~ (*μ*_*Z*_, 1) and *Z*_*j*_|(*Y *= - 1) ~ (0, 1), for *j *= 1,..., *q*.

We first consider the case of non-informative clinical covariates (*μ*_*Z *_= 0) and uncorrelated variables *X*_1_,..., *X*_*p*_, *Z*_1_,..., *Z*_*q*_, and consider the six following cases:

(null) p* = 0 (no informative molecular variables), for comparison

(a) p* = 5 and *μ*_*X *_= 0.5: few relevant variables, weak between-group shift

(b) p* = 5 and *μ*_*X *_= 0.8: few relevant variables, strong between-group shift

(c) p* = 50 and *μ*_*X *_= 0.3: many relevant variables, very weak between-group shift

(d) p* = 50 and *μ*_*X *_= 0.5: many relevant variables, weak between-group shift

(e) p* = 200 and *μ*_*X *_= 0.3: very many relevant variables, very weak between-group shift

To show that our method focuses on the *additional *predictive value of high-dimensional data, we also consider the following special setting (f): both the *q *= 5 clinical covariates and the p* = 5 relevant molecular predictors are highly predictive (*μ*_*Z *_= *μ*_*X *_= 1), but in the first case they are mutually uncorrelated (f.1), while we have *X*_1 _= *Z*_1_,..., *X*_5 _= *Z*_5 _in the second case (f.2).

For each setting, 100 simulated data sets are generated. The two following methods are applied to each data set for each setting:

A. Our method for *m*_stop _= 100, 500, 1000 and AIC-optimized *m*_stop _with *B *= 200 permutation iterations

B. Goeman's global test [7] with adjustment for the clinical covariates using the **globaltest **package [9]

### Simulation results

**Figure 2:**
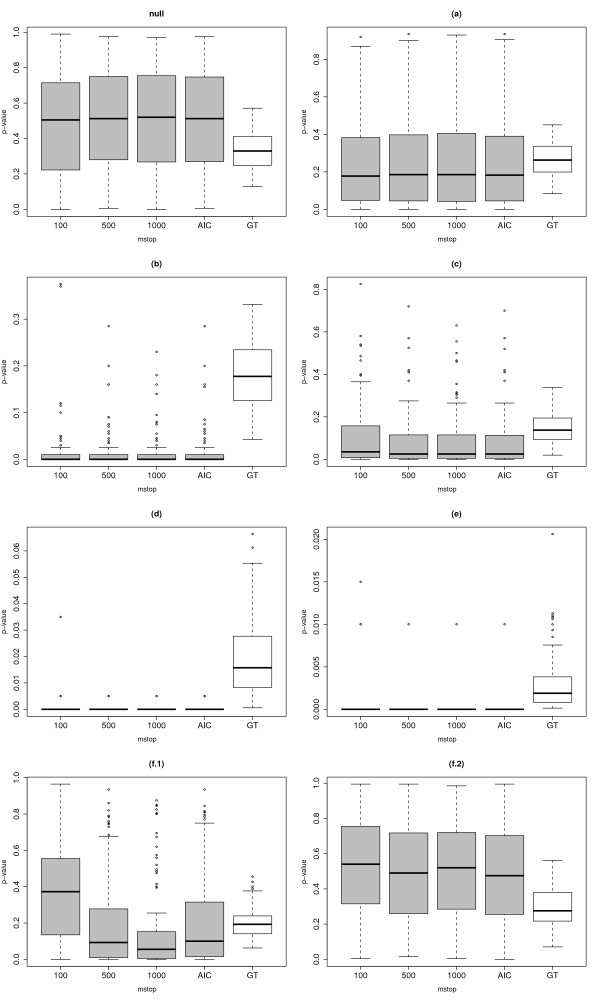
**Boxplots of p-values**. Boxplots of the p-values for the eight settings described in the Section 'Simulation design' using our new method with *m*_stop _= 100, 500, 1000 and AIC-optimized *m*_stop _(grey boxes) and using Goeman's global test (white boxes) for comparison.

Figure 2 represents boxplots of the p-values for the eight different settings. Three important results can be observed from the boxplots. Firstly, the influence of the parameter *m*_stop _seems to be minimal in all settings except in setting (f.1), where *m*_stop _= 1000 has a noticeably better power. Hence, this simulation study confirms that, as outlined in the Section 'The choice of *m*_stop_', the choice of *m*_stop _is not of crucial importance in most cases, and that *m*_stop _should rather be large. Secondly, our method shows high power in very different difficult situations such as a small number of strong predictors or a large number of very weak predictors. In all the examined settings, its power was in average better than the power of the standard globaltest - at the price of an increased computational expense. The power difference between our approach and the global test is especially striking in the case of a small number of strong predictors (b). Another interesting result is that the p-values of the global test are not uniformly distributed in the null case. Note, however, that we do not consider the permutation-based global test in this comparison study. Its results may be different with respect to the null-distribution of the p-values. Thirdly, our method finds additional predictive value in setting (f.1) but does not in setting (f.2) (i.e. when *X*_1 _= *Z*_1_,..., *X*_*q *_= *Z*_*q*_), thus fulfilling prerequisite 1.

### Real data analysis

We first analyze the ALL data set included in the Bioconductor package **ALL **[19]. The ALL data set is an expression set from a study on T- and B-cell acute lymphoblastic leukemia including 128 patients using the Affymetrix hgu95av2 chip with 12,625 probesets [20]. The data have been preprocessed using RMA. We consider the response remission/no remission, and the clinical covariates age, sex, T- vs. B-cell. After removing patients with missing values in the response or in the clinical covariates, we obtain a data set with 97 patients with remission and 15 patients without remission.

The second example data set considered in this paper is the van't Veer breast cancer data set [21]. The data set prepared as described in the original manuscript (only genes that show 2-fold differential expression and p-value for a gene being expressed < 0.01 in more than 5 samples are retained, yielding 4348 genes) is included in the R package **DENMARKLAB **[22], which we use in the article. The available clinical variables are age (metric), tumor grade (ordinal), estrogen receptor status (binary), progesterone receptor status (binary), tumor size (metric) and angioinvasion (binary).

**Figure 3:**
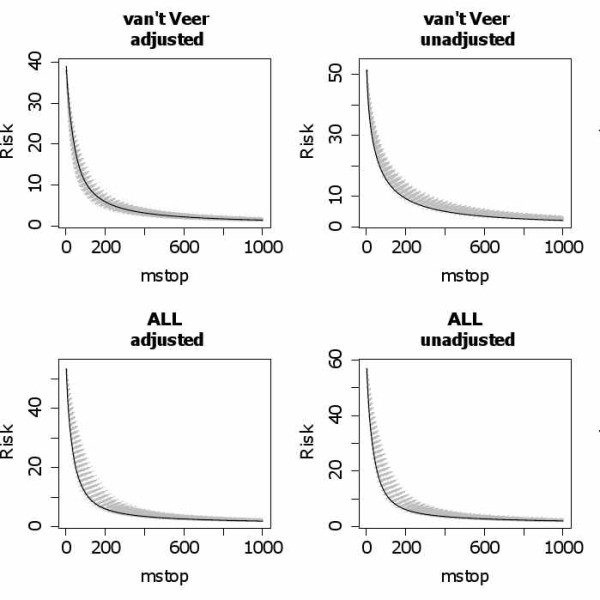
**Negative binomial log-likelihood in the real data study**. Negative binomial log-likelihood as a function of *m*_stop _for the original data sets (black) and for the permuted data sets (grey).

We apply the global test with adjustment for the clinical covariates and our new approach (with *m*_stop _= 100, 500, 1000 and AIC-optimized *m*_stop_) to both data sets. Additionally, we also apply the global test without adjustment and our method without first step (i.e. without adjustment for clinical covariates) for comparison. The results are given in Table 1. Whereas the ALL gene expression data seem to have additional predictive value, the van't Veer data do not, which corroborates previous findings [2,10]. A noticeable result of both Goeman's global test and our new approach is that the ALL data have more predictive value with adjustment than without adjustment, which may indicate that clinical and gene expression data are correlated and have contradictory effects on the response variable. In contrast, the van't Veer gene expression data seem to be marginally informative, but their predictive value vanishes when adjustment is performed. Figure 3 shows the negative binomial log-likelihood as a function of *m*_stop _for the original data sets (black) and for the permuted data sets (grey). It can be seen from Table 1 that different values of *m*_stop _may yield noticeably different results, in contrast to what was observed in simulations. In this context, the AIC-based choice of *m*_stop _is helpful.

**Table 1:** P-value obtained for real data sets

	global test			boosting-based	permutation test	
	adjustment		*m*_**stop **_= 100	*m*_**stop **_= 500	*m*_**stop **_= 1000	*m*_**stop **_AIC
ALL	yes	0.039	0.015	0.050	0.061	0.040
	no	0.078	0.013	0.068	0.136	0.025

van't Veer	yes	0.114	0.493	0.373	0.289	0.412
	no	0.015	0.006	0.009	0.010	0.009

## Discussion

### Good practice declaration

Our simulation and real data studies were performed with the values *m*_stop _= 100, 500, 1000 and with AIC-optimized *m*_stop _only. These values were chosen based on preliminary analyses in the vein of the Section 'The choice of *m*_stop_', but *not *based on the final results. The simulation settings were chosen based on short preliminary studies. The aim of these preliminary studies was to ensure informativeness in the sense that we avoided settings where all hypotheses are rejected (too strong predictors) or all hypotheses are accepted (too weak predictors). Following [23], the aim of the preliminary study was not to select the settings that would advantage our method compared to the concurrent globaltest approach. For reproducibility, the codes of the simulation and real data studies are available in the Additional files 1, 2 and 3. Our procedure is implemented in the package "globalboosttest" which is available from R-forge and will be sent to the CRAN as soon as possible.

### Variants of the two-step procedure

As suggested by a reviewer, other regularized regression techniques could be used in place of boosting regression in the second step of our procedure, for example, *L*_1_-penalized regression. Indeed, the Lasso and boosting regression can be seen as two sparse regularized regression methods addressing the same problem in a different way. If *L*_1_-penalized regression is applied in the second step, the penalty applied to the *L*_1 _norm plays the role of the number *m*_stop _of boosting steps as a complexity parameter. In principle, many other regularized regression techniques based on the logistic model may be used in the second step of our procedure, such as, e.g., *L*_2_-penalized regression. An extensive comparison study would go beyond the scope of this paper. However, a preliminary study using an arbitrary value for the penalty parameter indicates that similar performance can be obtained using *L*_1_-penalized regression as implemented in **glmpath **(data not shown).

Beside the high computational expense, an important problem of this approach is the choice of the penalty parameter. Whereas standard values of the number of boosting steps like *m*_stop _= 100 or *m*_stop _= 500 are expected to perform reasonably well in any case, there are no universal standard values for the penalty parameter in *L*_1_-penalized regression. Most importantly, the range of the penalty values considered by **glmpath **depends on the data. Thus, it may be difficult in practice to find a penalty value common to all permutations and the p-value cannot be simply calculated. On the whole, the choice of the complexity parameter seems to be more delicate in methods with direct penalization than in the context of boosting regression.

## Conclusions

We propose a simple boosting-based permutation procedure for testing the additional predictive value of high-dimensional data. Our approach shows good power in very different situations, even when a very small proportion of predictors are informative or when the signal in each informative predictors is very weak. Unlike approaches like pre-validation [24], it assesses the *additional *predictive value of high-dimensional data in the sense that the clinical covariates are involved in the model as a fixed offset. We provide clear advice for choosing the parameters involved in the procedure. The shrinkage factor *ν *should be set to the standard default value *ν *= 0.1 as recommended in previous publications [14]. The number *B *of permutations should be set as high as computationally feasible (the higher *B*, the more precise the p-value). The most delicate parameter is the number of boosting iterations *m*_stop_. Note, however, that the choice of *m*_stop _is not as crucial as in the context of prediction. The AIC-based procedure provides a reliable objective criterion. Except for the computational expense, there is almost no inconvenience to set the maximal number of boosting steps to a very large value, for instance  = 1000.

Note that our methodology can be easily generalized to a wide range of more complex regression problems such as survival analysis or non-linear regression. These problems can all be handled within the boosting regression framework using the **mboost **package [17,18]. Hence, our approach is essentially not limited to linear effects, although we focus on this special case in the present paper. The procedure can also be adapted to classification problems with asymmetric costs through the choice of an appropriate loss function. Another interesting and probably much more complex extension of this boosting-based procedure would be to perform individual tests to test the additional predictive value of each of the molecular variables. Since, especially for linear models, an efficient implementation of boosting is available [17], the computational effort of our procedure is manageable with standard hardware. Furthermore, the permutation procedure can be run in parallel which further reduces the required computing time [25].

## Authors' contributions

ALB drafted the paper and performed the analyses. Both authors developed the method and contributed to the manuscript. TH implemented the boosting procedure applied here. Both authors read and approved the final manuscript.

## Supplementary Material

Additional file 1 **R script for the analysis of the ALL data**. This R file includes the R script for reproducing our analyses of the ALL data. Click here for file

Additional file 2 **R script for the simulations**. This R file includes the R script for reproducing the simulation study. Click here for file

Additional file 3 **R function to generate the simulated data sets**. This R file includes the function used in our simulations to 1) generate the simulated data sets, 2) compute the p-value with our new method. Click here for file
